# Supplementary material for: Dynamics of Neutralizing Antibody and T-Cell Responses to SARS-CoV-2 and Variants of Concern after Primary Immunization with CoronaVac and Booster with BNT162b2 or ChAdOx1 in Health Care Workers
Source: Vaccines (Basel). 2022 Apr 19;10(5):639. doi: 10.3390/vaccines10050639 (PMC9147589; doi:10.3390/vaccines10050639)
Supplement: Supplementary file 1 [file vaccines-10-00639-s001.zip › vaccines-1676922-supplementary.pdf]

Table S1. Comparison of SARS-CoV2 surrogate viral neutralizing antibody (sVNT) (%inhibition) and seroconversion rate of sVNT ≥68% to wild type and VOC by multiplex sVNT in participants who received primary immunization with CoronaVac and booster with ChAdOx1 or BNT 162b2.

	ChAdOx1 booster<br>Median (IQR)	BNT162b2 booster<br>Median (IQR)	P	ChAdOx1 booster<br>N (%)	BNT162b2 booster<br>N%	P
Ancestral						
Post primary immunization week 4	57.5 (46.1-69.4)	67.3 (56.1-76.6)	0.07	8 (14.6)	23 (54.8)	0.02
Post primary immunization week 12	34 (26-44.9)	42.7 (34.1-57.4)	0.01	2 (3.6)	3 (7.1)	0.44
Post booster week 4	96.8 (91.5-98.5)	99.7 (99.4-99.8)	<0.001	52 (96.3)	42 (100)	0.21
Post booster week 12	84.8 (71.9-95)	95.6 (92.8-98.8)	<0.001	42/52 (80.8)	42 (100)	0.003
Alpha						
Post primary immunization week 4	49.4 (39.6-64)	62.2 (49.1-73)	0.03	6	19 (45.2)	0.03
Post primary immunization week 12	29.8 (23-39.1)	39.8 (29.5-49)	0.01	1 (1.8)	3 (7.1)	0.19
Post booster week 4	95.4 (87.2-98.2)	99.6 (99-99.8)	<0.001	93 (96.9)	51 (94.4)	0.25
Post booster week 12	81.1 (64.2-91.5)	95.2 (90.5-98.4)	<0.001	79 (84)	37 (71.2)	<0.001
Beta						
Post primary immunization week 4	38.2 (28.4-46.5)	48.5 (33.1-58.6)	0.01	0 (0)	6 (14.3)	0.08
Post primary immunization week 12	25.4 (19.4-29.9)	34.2 (24.6-39.5)	0.005	1 (1.8)	1 (2.4)	0.85
Post booster week 4	86.6 (75-93.2)	97.3 (95.7-98.1)	<0.001	43 (79.6)	42 (100)	0.002
Post booster week 12	64.9 (41.1-78.4)	88.3 (82.5-93.7)	<0.001	22 (42.3)	39 (92.9)	<0.001
Delta						
Post primary immunization week 4	54.2 (43.1-63.5)	60.2 (50.8-69.1)	0.07	3 (10.3)	13 (31)	0.05

Post primary immunization week 12	33.7 (24.7-42.2)	44.9 (33.2-52.7)	0.003	1 (1.8)	2 (4.8)	0.58
Post booster week 4	94.6 (85.5-98.3)	99.4 (98.9-99.6)	<0.001	52 (96.3)	42 (100)	0.50
Post booster week 12	77.9 (64.5-91)	94 (88.7-97.7)	<0.001	35 (67.3)	41 (97.6)	<0.001
Gamma						
Post primary immunization week 4	42.9 (35.7-54.5)	54.9 (39.1-62.4)	0.02	1 (3.5)	8 (19.1)	0.07
Post primary immunization week 12	26.9 (22.2-32.9)	36.1 (27-43.3)	0.001	1 (1.8)	2 (4.8)	0.58
Post booster week 4	87.7 (76.1-95)	97.9 (96.3-98.7)	<0.001	47 (87)	42 (100)	0.02
Post booster week 12	68.3 (45.8-79.5)	89.9 (84.7-94.7)	<0.001	26 (50)	39 (92.9)	<0.001
Omicron						
Post primary immunization week 4	21.6 (9.1-32.4)	24.1 (12.5-32.2)	0.72	0	0	NA
Post primary immunization week 12	24.6 (19.6-35.2)	31.5 (22-38.6)	0.03	0	0	NA
Post booster week 4	61.2 (44.3-71.3)	86.3 (73.8-93.6)	<0.001	19 (35.2)	35 (83.3)	<0.001
Post booster week 12	26.6 (12.4-37.7)	50.7 (34.6-71.9)	<0.001	3 (5.8)	12 (28.6)	0.004

Table S2. Comparison of T cell response by ELISpot to Pool S1 and S2 in participants who received primary immunization with CoronaVac and booster with ChAdOx1 or BNT 162b2.

	ChAdOx1 booster N=10 Median (IQR)	BNT162b2 booster N=10 Median (IQR)	P-value
Pool S1			
Pre immunization	6 (0-32)	16 (8-32)	0.42
Post primary immunization week 4	74 (44-140)	82 (44-152)	0.73
Post primary immunization week 12	40 (24-88)	28 (24-44)	0.54
Post booster week 4	106 (24-256)	162 (46-228)	0.65
Post booster week 12	52 (14-152)	148 (40-264)	0.25
Pool S2			
Pre immunization	8 (0-24)	14 (4-20)	0.51
Post primary immunization week 4	70 (44-188)	84 (40-128)	0.94
Post primary immunization week 12	56 (16-104)	56 (32-64)	0.77
Post booster week 4	94 (52-184)	146 (56-290)	0.45
Post booster week 12	58 (24-158)	104 (28-140)	0.82

Table S3. Factors associated with anti-SARS-CoV-2 spike total antibodies level after the booster dose

Factor	Univariate				Multivariate			
	Ratio	95%CI		P-value	Ratio	95%CI		P-value
Age (per year)	0.98	0.96	0.99	0.003	0.99	0.98	1.01	0.364
Female	1.14	0.76	1.71	0.54				
BMI (per kg/m^2)	0.99	0.95	1.03	0.738				
The interval between 2nd dose and a booster dose (per day)	1.04	1.03	1.06	<0.001	0.99	0.94	1.04	0.643
Vaccine type (BNT162b2 or ChAdOx1)	2.86	2.21	3.72	<0.001	3.61	1.11	11.76	0.03
Ant-SARS-CoV-2 total antibodies level prior to a booster (per 100 U/mL)	1.02	0.99	1.06	0.171	1.01	0.98	1.04	0.487

Using Random-effects GLS regression for anti-SARS-CoV-2 spike total antibodies (log transform)

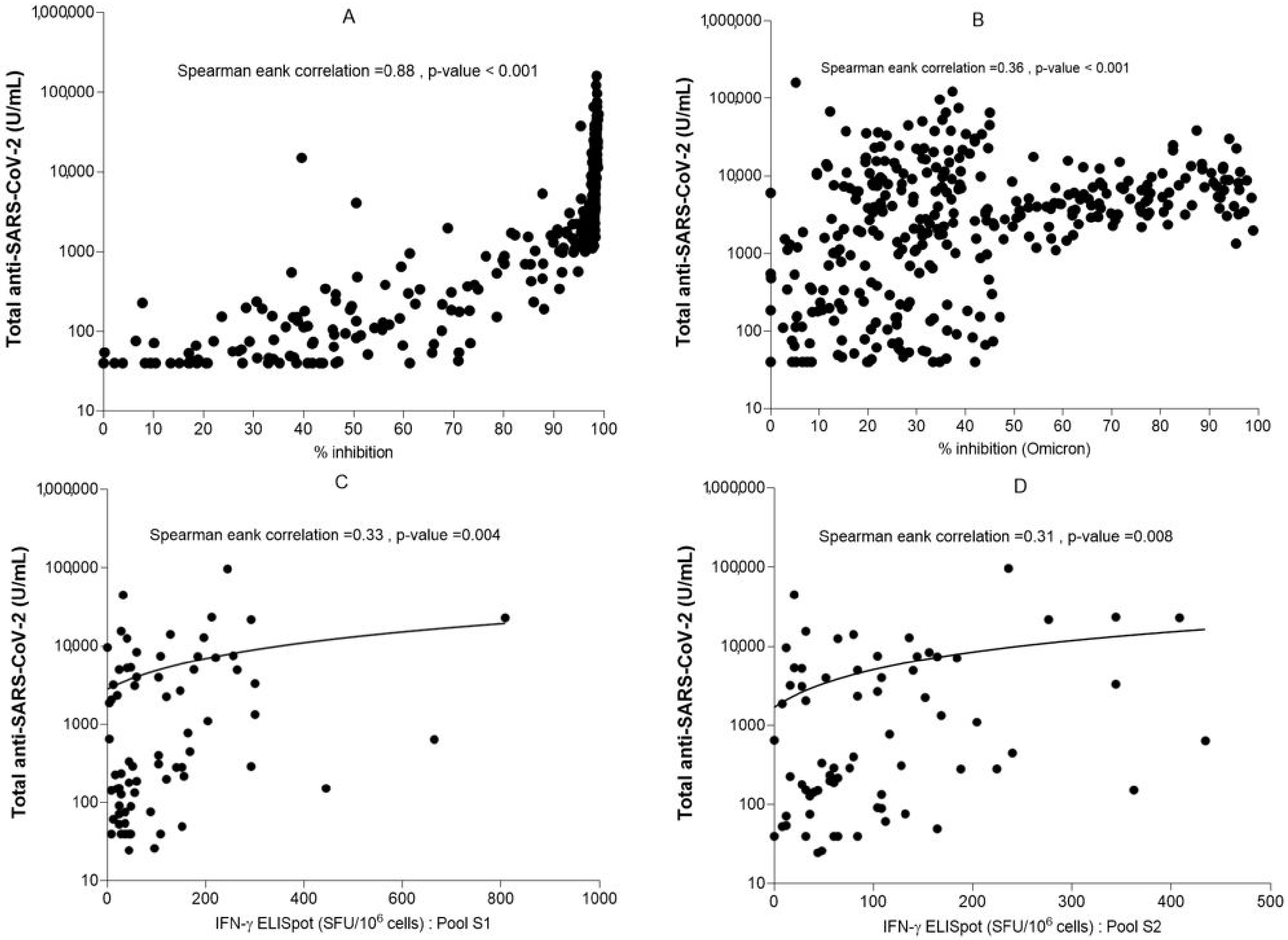

Figure S1. Correlation between total anti-SARS-CoV-2 and other immune response including (**A**) sVNT to ancestral type (**B**) sVNT to Omicron (**C**) the ELISpot of Pool S1 and (**D**) the ELISpot of S2.
